# MTHFR Ala222Val polymorphism and clinical characteristics confer susceptibility to suicide attempt in chronic patients with schizophrenia

**DOI:** 10.1038/s41598-020-57411-1

**Published:** 2020-03-19

**Authors:** Jia Hong Liu, Cheng Zhu, Ke Zheng, Wei Tang, Li Li Gao, Tammy H. Trihn, Hanjing Emily Wu, Da Chun Chen, Mei Hong Xiu, Xiang Yang Zhang

**Affiliations:** 1 Wenzhou Kangning Hospital, Wenzhou, China; 2 Department of Psychiatry and Behavioral Sciences, The University of Texas Health Science Center at Houston, Houston, TX USA; 3 Beijing HuiLongGuan Hospital, Peking University, Beijing, China; 4 CAS Key Laboratory of Mental Health, Institute of Psychology, Chinese Academy of Sciences, Beijing, China

**Keywords:** Schizophrenia, Disease genetics

## Abstract

Patients with schizophrenia (SCZ) exhibit higher suicide rates than the general population. However, the molecular mechanism responsible for the high rate of suicidal behavior in SCZ remains poorly understood. *MTHFR* Ala222Val (C677T; rs 1801133) polymorphism has repeatedly demonstrated to play a pathological role in numerous mental disorders, but none of these studies focused on the susceptibility of suicidal behavior in SCZ. In the present cross-sectional study, we recruited 957 chronic inpatients with SCZ and 576 healthy controls to assess the psychopathological symptoms of SCZ and compare the frequency of the *MTHFR* Ala222Val genotype in both suicide attempters and non-attempters. Our results demonstrated no significant differences in *MTHFR* Ala222Val genotype and allele distributions between the SCZ patients and controls (p > 0.05), but showed a statistical significance in the distribution of Ala/Val genotype between suicide attempters and non-attempters (p < 0.05). Further logistic regression analysis showed that *MTHFR* Ala222Val genotype, psychopathological symptoms, number of cigarettes smoked per day and drinking status were related to suicide attempts in SCZ (p < 0.05). Our study demonstrated that *MTHFR* Ala222Val polymorphism and some clinical characteristics might confer susceptibility to suicide in patients with SCZ.

## Introduction

Patients with schizophrenia (SCZ) are at high risk for attempting and completing suicide. Suicide is characterized by intentional termination of one’s life, which is an important sequelae of chronic schizophrenia^1,2^. To date, suicide has been considered one of the most common causes of premature death in SCZ patients^3^. The average life expectancy of patients with SCZ is estimated to have decreased by approximately 14.6 years by suicide^4^. However, the pathophysiological mechanisms for the suicidality of SCZ patients are still unclear, particularly the biological pathological mechanism.

Evidence from twins, adoption, and family studies showed a significant increase of suicidal rates in suicidal probands^5^, which suggests that genetic risk factors may play an important role in suicidal behavior. The heritability of suicidal behavior is about 45%, while environmental components account for 57%^6^. Based on earlier genetic association studies focusing on several biological systems associated with suicidal attempts, a variety of potential candidate genes have been identified. For example, brain-derived neurotrophic factor (BDNF) and its receptor (NTRK2), which are highly expressed in the prefrontal cortex and hippocampus, have been shown as strong candidates for suicide; however, these results are controversial^6^. Also, another biological system involved in serotonergic transmission has been found to be candidate genes for suicidal behavior, including a tryptophan hydroxylase involved in synthesis, monoamine oxidase A involved in degradation, and a serotonin-1-A receptor involved in transmission^7^. Studies have demonstrated that nearly 90–98% of suicides are committed by people with some type of mental illness^8^. Thus, mental illness can be identified as the strongest risk factor for suicide. Among them, SCZ is one of the most common diseases associated with suicidal behavior^9^. Above evidences suggest that overlapping genetic risk factors are associated with suicidal behavior and psychopathology. However, genetic markers associated with the etiology and pathophysiology of suicidal behavior in psychiatric patients, especially SCZ patients, are still unknown.

The *MTHFR* gene, located on chromosome 1 encodes a 5-methylenetetrahydrofolate reductase that catalyzes the reduction of 5,10-methylenetetrahydrofolate (methylene THF). Impaired MTHFR metabolism leads to hypomethylation of DNA, which increases the mutation ratio and leads to chromosome instability^10^ in addition to increased concentrations of potentially toxic homocysteine (Hcy) and abnormal concentrations of neurotransmitters^11^. It was found that the substitution (C to T) of nucleotide 677 (rs1801133) in exon 4 of the *MTHFR* gene resulted in amino acid substitution (Ala222Val) and a reduction of MTHFR activity, which was most frequently studied in relation to mental disorders including SCZ^12–14^, autism spectrum disorders^15–17^, affective disorders^18–20^, anxiety disorders^21,22^ and alcohol dependence^23^. To the best of our knowledge, only one study has investigated whether the *MTHFR* Ala222Val polymorphism was associated with suicide in 692 victims of completed suicide and 3257 healthy controls. No association was found between the *MTHFR* Ala222Val polymorphism and suicide^24^. In the study, only 16 of the 692 subjects were diagnosed with SCZ, and the diagnoses were based upon medical and forensic examinations of post-mortem rather than evaluations by psychiatrists. Recently, several striking meta-analyses have demonstrated that *MTHFR* Ala222Val polymorphism is significantly associated with SCZ^13,25–28^. In particular, three meta-analyses have shown a positive association between *MTHFR* Ala222Val polymorphism and SCZ in Asian population, but not in the European population^13,28,29^. Furthermore, the SCZ patients with the *MTHFR* Val/Val or Ala/Val genotype were found to have higher Hcy levels than those with the Ala/Ala genotypes, which might be associated with the increased risk of SCZ^30,31^. A recent meta-analysis also indicated that increased plasma total Hcy levels may be associated with a higher risk of SCZ^27^. Interestingly, one study showed that *MTHFR* Val/Val genotype affected gray matter density and memory impairment in SCZ patients in a Chinese population^32^. Taken together, these studies have suggested that the *MTHFR* gene polymorphisms, especially the *MTHFR* Val/Val genotype, may be involved in the psychopathology of suicidality in SCZ patients.

Considering the high suicide rate of SCZ patients, the pathogenic role of the *MTHFR* gene, and the higher Hcy  level in SCZ, it would be interesting to investigate the relationship between the functional polymorphism of *MTHFR* Ala222Val and suicide attempt of SCZ patients in a genetically more homogeneous Han Chinese population. Based on previous literature, we hypothesized that the Val allele carriers of the *MTHFR* Ala222Val would be associated with increased risk for suicide attempt in SCZ and may interact with some relevant clinical risk factors. We then examined whether the interaction of genetic and environmental factors might affect the risk of attempted suicide in SCZ patients.

## Results

The demographic and clinical characteristics are shown in Tables 1 and 2. There were significant differences in sex, age, smoking status, and BMI between patients and healthy controls (all p < 0.01), which were controlled in the following analyses as confounding factors. The Ala222Val allelic and genotypic frequencies of SCZ patients and healthy controls are shown in Table 1. Distributions of the *MTHFR* Ala222Val genotypes were consistent with the Hardy-Weinberg equilibrium in both SCZ patients and healthy controls (both p > 0.05). Further, there were no significant differences in the Ala222Val allelic and genotypic distributions between SCZ patients and healthy controls (both p > 0.05). After adjusting for sex, smoking, BMI, and age, we still did not find any differences in  the Ala222Val allelic and genotypic frequencies between the two groups (both p > 0.05). In addition, we did not find any association between the *MTHFR* Ala222Val genotypes and the positive and negative syndrome scales^33^ (PANSS) total and its 3 subscale scores (all p > 0.05).

**Table 1:** Demographic, clinical and genetic data in patients with schizophrenia and healthy controls.

Variable	SCZ (n = 957)	HC (n = 576)	F or *χ*^2^ (p)
Gender(male/female), n	783/174	263/313	216.8 (<0.001)
Age (years)	47.8 ± 10.2	45.8 ± 12.8	11.7 (<0.01)
Education (years)	9.3 ± 6.4	8.7 ± 3.2	1.5 (0.09)
Smokers/Non-smokers, n	627/318	218/358	117.7 (<0.000)
BMI (kg/m^2^)	24.5 ± 3.9	25.1 ± 3.9	2.7 (<0.01)
Drinkers/Non-drinkers, n	137/727	121/455	6.2 (0.013)
*MTHFR* genotype distribution			2.0 (0.36)
CC n(%)	298 (38.9)	145 (34.8)	
CT n(%)	344 (45.0)	202 (48.4)	
TT n(%)	123 (16.1)	70 (16.8)	
***MTHFR*** **allele frequency**			
C/T, n	590/940	342/492	0.08 (0.83)

Note: Data is given as mean ± standard deviation; BMI = body mass index; SCZ = schizophrenia; HC = healthy control; MTHFR = Methylenetetrahydrofolate reductase.

**Table 2:** Demographic, clinical and genetic data in patients with schizophrenia divided with regard to suicide attempts.

Variable	Attempters (n = 125)	Non-attempters (n = 642)	F or χ^2^ (p)
Gender (male/female), n	105/20	522/120	0.51 (0.48)
Age (year), n(%)			4.2 (0.13)
≦30	11 (8.8)	36 (5.6)	
30–60	91 (72.8)	442 (68.8)	
≧60	23 (18.4)	164 (25.6)	
Education (year)	8.9 ± 2.9	9.3 ± 6.9	0.75 (0.45)
Drinkers/Nondrinkers, n	35/90	91/676	23.1 (0.000)
Age of smoking onset (year)	19.2 (3.1)	21.9 (4.0)	2.51 (0.015)
Smoking status, n(%)			0.52 (0.47)
Smokers	81 (64.8)	394 (61.4)	
Non-smokers	44 (35.2)	248 (38.6)	
Marital status, n(%)			3.1 (0.38)
Single	84 (67.2)	381 (59.3)	
Married	22 (17.6)	130 (20.2)	
Divorced	18 (14.4)	122 (19.0)	
Widowed	1 (0.8)	9 (1.4)	
BMI (kg/m^2^)	24.4 ± 3.9	24.6 ± 4.0	0.46 (0.65)
Age of illness onset (year)	23.7 (6.5)	23.5 (5.5)	0.31 (0.76)
Duration of illness (year)	24.8 ± 9.9	21.1 ± 9.9	4.2 (0.000)
Antipsychotic dose (CPZ)	472.3 ± 536	452.2 ± 392	0.28 (0.60)
**PANSS factors (score)**			
Positive	8.7 ± 74.8	7.7 ± 4.9	4.7 (0.03)
Negative	19.2 ± 7.6	18.9 ± 6.4	0.15 (0.70)
Cognitive	7.5 ± 3.0	8.1 ± 3.5	3.5 (0.06)
Depression	9.2 ± 5.2	7.9 ± 5.2	7.4 (0.000)
Excitement	4.9 ± 2.2	4.6 ± 1.7	4.2 (0.04)
*MTHFR* allele distribution, n(%)			0.09 (0.83)
Ala	153 (62.3%)	787 (61.3%)	
Val	97 (37.7%)	497 (38.7%)	

Note: Data is given as mean ± standard deviation; BMI = body mass index; PANSS = Positive and negative syndrome scale; CPZ = Chlorpromazine equivalent dose; MTHFR = Methylenetetrahydrofolate reductase.

The SCZ patients included 152 (15.9%) suicide attempters and 805 non-attempters. The Ala222Val allelic and genotypic frequencies of suicide attempters and non-attempters of patients are shown in Tables 2 and 3. On the level of genotypes, there was a significant difference between attempters and non-attempters (*χ*^2^ = 4.7, df = 2, p = 0.039), and the Ala/Val genotype was less frequent in attempters than in non-attempters (36.1% vs 46.6%) in the codominant models (Ala/Val vs. Val/Val). The *MTHFR* Ala/Val genotype was associated with a lower suicide risk compared to the Val/Val genotype (OR = 0.36, 95% CI = 0.23–0.59, adjusted p = 0.000; Table 3) in patients  with SCZ. There was no significant difference in the distribution of alleles between the two groups (*χ*^2^ = 0.09, df = 1, p = 0.83).

**Table 3:** Multiple logistic regression analysis of the *MTHFR* Ala222Val polymorphism and suicide susceptibility.

Genotype	Attempers n = 125	Nonattempters n = 642	p-value^a^	Crude OR (95% CI)	Adjusted OR (95% CI)	p-value^b^
***MTHFR*** **Ala222Val (HWE** = **0.791)**						
Val/Val	26	99		1	1	
Ala/Val	45	299	**0.039**	0.61 (0.35–1.01)	0.36 (0.23–0.59)	**0.0000**
Ala/Ala	54	244	0.52	1.10 (0.64–1.87)	0.97 (0.73–1.28)	0.83

Notes: ^a^Chi-squared tests were used to determine differences in genotype distributions between attempters and nonattempters.

^b^Adjusted for age, gender, smoking, BMI, drinking and marital status.

We further analyzed the characteristics related to suicide and found significant differences in drinking status (p < 0.001), onset age of smoking (p < 0.05), cigarettes smoked each day (p < 0.05), duration of illness (p < 0.01), positive factor of PANSS (p < 0.05), depression factor (p < 0.001), and excitement factor (p < 0.05) between attempter and non-attempter groups, as shown in Table 4. However, only the difference in drinking status and depression factor of PANSS remained significant after the Bonferroni correction test (corrected p < 0.05). There was no significant difference between the suicide attempters and non-attempters in age, gender, BMI, age of onset, marital status, and antipsychotic types and doses (in chlorpromazine equivalents) (all p > 0.05).

**Table 4:** Demographic and clinical data in patients with schizophrenia divided with regard to suicide attempt and *MTHFR* Ala222Val genotypes.

Variable	Attempter			Nonattempter		
	Ala/Ala	Ala/Val	Val/Val	Ala/Ala	Ala/Val	Val/Val
Gender (male/female),n	32/6	59/5	17/5	212/48	240/40	82/19
Age (year)	45.6 ± 9.7	45.6 ± 9.7	47.1 ± 10.1	49.0 ± 9.4	48.1 ± 9.3	47.9 ± 9.6
Education (year)	9.3 ± 2.3	7.9 ± 2.5	8.6 ± 2.6*	9.3 ± 2.5	9.3 ± 5.8	8.5 ± 2.5
Drinkers/Nondrinkers, n	3/18	20/23	11/39*	13/76	31/243	34/189
Cigarette/day	27.4 ± 5.6	23.9 ± 4.5	21.3 ± 3.8*	22.5 ± 4.8	19.5 ± 10.3	18.8 ± 2.0
Smokers/Non-smokers, n	17/7	32/13	36/18	62/37	180/118	167/76
BMI (kg/m^2^)	24.9 ± 3.9	25.4 ± 4.3	23.6 ± 3.7	24.7 ± 4.1	24.6 ± 3.9	24.6 ± 3.9
Age of onset (year)	25.0 ± 9.3	23.1 ± 4.8	23.8 ± 6.3	23.4 ± 6.0	23.5 ± 5.4	23.2 ± 4.8
Duration of illness (year)	20.0 ± 8.9	22.3 ± 8.7	23.4 ± 9.6	25.6 ± 9.2	24.6 ± 9.8	24.6 ± 9.1
Antipsychotic dose (CPZ)	459.5 ± 464.0	402.9 ± 175.4	509.7 ± 403.2	446.9 ± 311.9	478.0 ± 448.7	421.3 ± 270.1
**PANSS factors (score)**						
Positive	8.7 ± 4.8	8.8 ± 4.1	8.9 ± 5.1	7.9 ± 4.9	7.4 ± 4.9	7.0 ± 4.3
Negative	19.6 ± 5.2	18.8 ± 6.5	20.9 ± 6.3	3.6 ± 1.2	3.5 ± 1.2	3.6 ± 1.2
Cognitive	7.7 ± 3.0	7.5 ± 2.6	8.4 ± 3.1	8.2 ± 3.5	7.9 ± 3.6	8.1 ± 3.4
Depression	5.0 ± 2.6	4.3 ± 1.9	4.7 ± 2.3	3.6 ± 1.2	3.5 ± 1.2	3.6 ± 1.2
Excitement	4.6 ± 0.9	5.1 ± 2.7	5.2 ± 2.5	4.7 ± 2.0	4.6 ± 1.8	4.6 ± 1.5

Note: Data is given as mean ± standard deviation; BMI = body mass index; PANSS = Positive and negative syndrome scale; CPZ = Chlorpromazine equivalent dose; MTHFR = Methylenetetrahydrofolate reductase.

*p < 0.05.

In the binary logistic regression model for associations with suicide attempt, education (OR = 0.89, 95% CI [0.82, 0.98], Wald *χ*^2^ = 5.6, p = 0.018), PANSS depression factor (OR = 1.08, 95% CI [1.03, 1.13], Wald *χ*^2^ = 11.04, p = 0.001), number of cigarettes smoked per day (OR = 1.20, 95% CI [1.18, 1.52], Wald *χ*^2^ = 4.9, p = 0.043), and drinking status (OR = 0.40, 95% CI [0.24, 0.66], Wald *χ*^2^ = 13.0, p < 0.001) remained significant and *MTHFR* Ala222Val polymorphism (OR = 1.72, 95% CI [0.94, 3.2], Wald *χ*^2^ = 3.1, p = 0.07) approached significance.

The whole sample in the current study had a power of 0.99 to detect the association of *MTHFR* Ala222Val with SCZ as dominant, recessive, and log additive inheritance, with an OR of 2 (α = 0.05, two-tailed test). Moreover, the suicide attempt sample had 0.88–0.99 statistical power to detect this polymorphism to have relationship with suicide attempts as dominant, recessive, and log additive inheritance, with an OR of 2 (α = 0.05, two-tailed test).

## Discussion

There are three main findings in the present study. First, we did not find any significant difference in the *MTHFR* Ala222Val polymorphism between the patients with SCZ and healthy controls, which was in accordance with a couple of previous studies^34–36^, suggesting that the Ala222Val polymorphism of the *MTHFR* gene did not contribute to increased risk of SCZ in our Chinese samples. Second, the *MTHFR* Ala222Val polymorphism appears to be weakly correlated with suicide attempts in SCZ. Moreover, compared to patients without lifetime suicide attempts, the Ala/Val genotype was significantly less frequent and the Val/Val genotype was more frequent in lifetime suicide attempts of SCZ patients (35.8% vs 46.6%, 20.3% vs 15.4%). Third, in addition to *MTHFR* Ala222Val polymorphism, higher psychopathological symptoms and more severe depressive symptoms, more cigarettes smoked, and more drinking were significantly correlated to suicidal risk, suggesting that a multifactorial etiology involving both genetic heritability and environmental factors may contribute to suicidal behaviors among SCZ patients.

Numerous studies have investigated the possible role of functional polymorphism of *MTHFR* gene in the risk of pathogenesis of SCZ, but the results are inconsistent. We found that *MTHFR* Ala222Val polymorphism did not play a significant role in the pathogenesis of SCZ in our current study, which is consistent with several previous studies in Iranian, Korean, and Nordic populations^37^. However, the most recent meta-analysis reported a positive association between Ala222Val and SCZ in eastern Asia^27^, which is completely different from our results. A possible reason for the inconsistencies in these genetic association studies may be due to ethnic background. For example, the genotype frequency distribution of Ala222Val is significantly different between the Chinese and European populations. The frequency of Val/Val genotype in healthy controls was approximately 16% in the Chinese population, but only about 9% in the European population. Therefore, interethnic differences in the genotype frequencies of the *MTHFR* Ala222Val polymorphism may lead to inconsistent results across the different populations. Other factors may also contribute to these controversial results, such as small or moderate genetic effects of Ala222Val, diet with increased folate consumption, relatively small sample sizes, heterogeneity in SCZ diagnosis, and gender stratification.

To our best knowledge, this is the first study to report the positive association between the *MTHFR* Ala222Val polymorphism and suicidal  behavior in SCZ patients. Moreover, no significant association was found between the *MTHFR* Ala222Val genotypes and the PANSS total and its 3 subscale scores (all p > 0.05), suggesting that the *MTHFR* Ala222Val polymorphism appears not to be a general marker of a more severe disease but rather specific for suicide. Only one previous study investigated the association between suicide and Ala222Val polymorphism in postmortem and reported contradictory results^24^. A possible explanation for the inconsistency in results is that the subjects enrolled in the present study were suicide attempters and not suicide completers. In addition, different methods were used in the diagnosis of SCZ (long-term chronic hospitalization of SCZ patients vs no psychological autopsy). Thus, the differences in the participants may account for the discordant results in these two studies. Our current study suggested that *MTHFR* Ala/Val genotype may lay a critical role in suicidal behaviors. The 222Val allele was considered to be a mutant allele. Ala222Val polymorphism was thought to be associated with alteration of MTHFR enzyme activity as the heterozygous genotype (Ala/Val) and mutant homozygous genotype (Val/Val) have only 67% and 25% of the MTHFR enzyme activity of their Ala/Ala wild type counterparts, respectively^22^. In the present study, the Val/Val genotype was over-present and the Ala/Val genotype was less present among suicide attempters in patients with SCZ. We speculate that the association of *MTHFR* Ala/Val genotype with suicide attempt may be in relation to the reduced MTHFR enzyme activity. A reduction in MTHFR enzyme activity results in a decline in its ability to irreversibly reduce 5,10-methylene THF to 5-methyltetrahydrofolate (5-methyl THF) and produce methyl groups involved in epigenetic regulation^22^. The declined enzyme activity may either raise the concentration of potentially toxic Hcy in those with inadequate dietary folate intake, or lead to hypomethylation of DNA^38,39^. The studies in *MTHFR* gene knockout mice have shown that plasma total Hcy was 10-fold and 1.6-fold higher, respectively, in MTHFR -/- and MTHFR +/− mice than in wild-type controls^40,41^. Numerous studies have also demonstrated that elevated total Hcy levels in plasma are one of the most important risk factors for psychiatric diseases such as depression and SCZ^42–45^, although few studies have focused on the direct relationship between suicide attempt and Hcy in SCZ. However, it is worth mentioning that this explanation for the association between heterozygous genotypes and suicide attempts may stem from the enzymatic effects of genotypes, which is only our speculation. If our interpretation in this part was of major importance, there should be an even stronger difference between the wildtype (Ala/Ala) and the homozygous mutant genotype (Val/Val) than between Ala/Ala and Ala/Val genotypes, and the difference should also be obvious when alleles were compared. However, the fact is that the Ala/Ala and Val/Val genotypes were almost equally present and far from significantly different after clinical variables were controlled  (Table 3). Moreover, there was no significant difference in allele frequencies between attempters and non-attempters. Taken together, this interpretation for the association between heterozygous genotypes and suicide attempts due to the enzymatic effects of genotypes is quite speculative and may provide only part of the reasons. However, there are some other important mechanisms underlying the association between heterozygous genotypes and suicide attempts; for example, proximal variants or genes that may be in linkage disequilibrium with the *MTHFR* Ala/Val polymorphism may also be associated with suicide attempt in schizophrenia. Therefore, the mechanism linking the *MTHFR* Ala/Val gene polymorphism and attempted suicide is still unknown, which warrants further investigation.

It is well known that the severity of depressive symptoms was the most robust risk factor of suicidal behaviors in psychiatric disease^46,47^. Accordingly, a study of long-term hospitalized SCZ patients in China found that 72% of suicidal attempts were accompanied by depressive symptoms^48^. Further, the 222Val/Val genotype was found to be associated with depression, anxiety, and cognitive impairment, which all had close relationships with suicidal behavior in later life^49^. Since the interaction of Hcy and depressive symptoms play a pathogenic role in suicidal behaviors, we presumed that increased Hcy, as a consequence of the mutant genotype in embryonic neural cells, exerts neurotoxic effects, resulting in neonatal neurodevelopmental defects related to suicidal behavior^50,51^. Indeed, studies found that high Hcy levels lead to abnormal levels of monoamine neurotransmitters, such as 5-serotonin and dopamine and cause aberrant BDNF levels^52–54^, which may all participate in the occurrence of depressive symptoms related closely to suicide behavior in SCZ. On the other hand, abnormal epigenetic modifications caused by dysfunctional enzyme activity during pregnancy, such as DNA hypomethylation, are increasingly recognized to have lasting developmental implications with regard to suicide behavior^55,56^. Evidence supports that hypomethylation of DNA could increase the mutation ratio and cause chromosome instability with neurodevelopmental consequences due to downstream effects on dopaminergic and serotonergic systems important for developmentally stable emotional, cognitive, and behavioral phenotypes in childhood and may increase suicidal behaviors later in life^57^. However, it is worthy of mentioning that although there was association between the 222Val/Val genotype and depression, anxiety, and cognitive impairment, which are conditions associated with suicidal behavior^49^, our results indicated association of attempted suicide with the Ala/Val genotype but not with Val/Val genotype, suggesting a heterozygous effect of the *MTHFR* Ala222Val gene polymorphism on suicidal behavior. Interestingly, a recent study reported that Ala/Val genotype of *MTHFR* Ala222Val gene polymorphism was significantly more frequent in healthy controls than patients with depression, suggesting that a heterozygous effect of the *MTHFR* Ala222Val gene polymorphism may be a protection from depression^56^. To our knowledge, this is the first report to show the heterozygous effect of the *MTHFR* Ala222Val gene on the clinical variability of schizophrenia phenotype. Alternatively, the effects of Ala222Val polymorphism on attempted suicide may also be due to linkage disequilibrium between this polymorphism and a functional polymorphism in or near the *MTHFR* gene. Therefore, in order to fully understand the relationship between *MTHFR* genetic variants and suicide attempt in schizophrenia, haplotype analysis combining multiple *MTHFR* genetic polymorphisms in larger samples will be needed.

Both SCZ and suicide behavior have a multifactorial and complex etiopathogenesis, arising from a combination of genetic and environmental factors^58,59^. We found that more symptoms (positive symptom, depressive, excitement factors), longer duration of illness, alcohol consumption, and more cigarettes smoked each day were associated with suicidal behavior in SCZ, suggesting important environmental factors in suicide behavior among SCZ patients.

There are several limitations that should be noted. First, the effect of *MTHFR* Val/Val genotype can be alleviated by increasing the consumption of folate and, to a degree, cobalamin^60^. However, we did not measure plasma folate and homocysteine levels in the subjects and did not have information on whether our subjects lacked folate food fortification or not. Second, there was a significant difference in the age between suicide attempters and non-attempters. Many studies have found that the common factors which may exacerbate folate deficiency are older age, male sex, and alcoholism^61–63^. Third, there were more female subjects in the SCZ group, which may have led to bias in the statistical analysis due to the imbalance in the number of subjects in each sex category in our current study. Fourth, it should be noted that we only found a weak association between the Ala222/Val genotype and suicide attempt in SCZ.

To summarize, in the present study, we showed no relationship between *MTHFR* Ala222/Val polymorphism and  SCZ, but a weak association between this polymorphism and suicide attempt in SCZ. Moreover, psychological symptoms, drinking status, and smoking possibly interacted with the *MTHFR* gene variant and appeared to play an important role in suicide attempts among SCZ patients. However, considerable caution should be noted in the interpretation of the results. Since the sample size was small, the significant association between *MTHFR* Ala222/Val polymorphism and suicidal behavior may be due to multiple tests or small sample size. Further studies in a larger sample of various ethnic populations are unavoidable to investigate the interactions between these genes and the environment.

## Methods

### Subjects

957 chronic inpatients with SCZ were recruited from two large psychiatric hospitals in Northern China. The inclusion criteria were as our prior studies: age 20–75 years; Han Chinese; and diagnosis of SCZ according to DSM-IV by the trained psychiatrists. All patients were of the chronic type with an illness duration of at least 5 years. Also, they had been taking stable doses of oral antipsychotic medication for at least one year prior to recruitment. Antipsychotic medication consisted mainly of prescribed drug monotherapy, including clozapine (n = 430), risperidone (n = 210), chlorpromazine (n = 69), sulpiride (n = 49), perphenazine (n = 45), quetiapine (n = 42), haloperidol (n = 34), aripirazole (n = 29), and others (n = 49). Average of antipsychotic dose (in chlorpromazine equivalents) was 438 ± 407 mg/day.

576 unrelated healthy controls were recruited through advertisement from local communities near the hospital during the same period as the patients. A research psychiatrist ruled out potential controls with Axis I disorders on DSM-IV at the present or in their lifetime after a clinical structured interview. All the healthy controls were Chinese Han population.

We carried out a physical examination and laboratory tests from all subjects. Any subjects with abnormalities in physical health were excluded. All subjects gave informed consent to participate in the study, which was approved by the Institutional Review Board of Beijing HuiLongGuan hospital. In addition, all methods were performed in accordance with Declaration of Helsinki promulgated by the National Institute of Health.

### Clinical measures

PANSS was used to assess symptoms of SCZ on the same day of blood drawing. Prior to the assessment, four psychiatrists received training sessions in  the use of PANSS and conducted repeated assessment tests. After training, inter-rater correlation coefficient was greater than 0.8 for the PANSS total score.

In this study, a five-factor model of SCZ symptoms was utilized, labeling symptoms as ‘positive’, ‘negative’, ‘cognitive’, ‘depression’, and ‘excitement’^64^. The new positive factor was composed of P1, P3, P5, and G9, and the new negative factor was composed of N1, N2, N3, N4, N6, and G7. In addition, the three items P2, N5, and G11 of the PANSS scale constitute a cognitive factor, G2, G3, and G6 for a depressive factor, and P4, P7, G8, and G14 for an excitement factor.

History of suicide attempts were assessed by the same four psychiatrists based on record reviews and interview data according to a definition by WHO^65^. In this study, suicide attempts were defined as deliberate self-destructive behavior, with at least some intention to kill themselves, but not causing death^65^. During the study interview, the main question was “Have you tried to commit suicide in your lifetime?” If the answer was yes, then all subjects were asked about previous suicide attempts, including the following details: number of attempts, exact date and method of each suicide attempt. Totally, there were 152 patients who had a history of suicidal attempt (attempters) and 805 patients without history of suicidal attempt (non-attempters). The average number ± standard deviation of attempted suicide among attempters was 1.51 ± 0.94 times (ranging from 1 time to 5 times).

### MTHFR polymorphism analysis

Based on previous studies^14,24^, we selected one functional single nucleotide polymorphism Ala222Val in the promoter of *MTHFR* gene. The polymorphism was genotyped following the standard procedure as described in Sequenom Genotyping Protocol and the previous studies^66^. The primers and extent probe of *MTHFR* Ala222Val were: sense: 5′-ACGTTGGATGCTTGAAGGAGAAGGTGTCTG-3′, antisense: 5′-ACGTTGGATGCTTCACAAAGCGGAAGAATG-3′, and probe: AAGGTGTCTGCGGGAG. The sample genotyping success rate of this study averaged 98.9%. 5% of all DNA samples were repeated, showing 99.5% reproducibility for SNP results.

### Statistical analysis

Deviations from Hardy-Weinberg (HW) equilibrium were tested by using the *χ*^2^ test for goodness of fit. The differences in allele and genotypic frequencies of *MTHFR* Ala222Val polymorphism between groups were analyzed by using the *χ*^2^ test. Further, we used binary logistic regressions to calculate odds ratios (*ORs*) and their 95% confidence intervals (CIs) to evaluate the effects of different genotypes on SCZ or suicide after controlling for the confounding factors.

Analysis of variance (ANOVA) for continuous variables and the chi-squared test for categorical variables were used to analyze the differences between groups (patients vs. healthy controls or suicide attempters vs. non-attempters). The relationship between the variables is evaluated using Pearson’s correlation coefficient. Bonferroni correction was used with each analysis to correct multiple tests. The variables that were significantly correlated with suicide attempt were included in the next logistic regression analysis, which was carried out to adjust for confounding factors. In the logistic regression model, the dependent variable was suicide, and the independent variables were those that were significantly different between suicide attempters and non-attempters.

We used SPSS version 15.0 to perform all statistical analysis, with two-tailed p values of less than 0.05. The power of the sample was calculated using Quanto software, with the relative risk and known risk allele frequencies under dominant models, respectively.
